# Revealing the evolution of behavioral activation research trends over two decades through keyword network analysis

**DOI:** 10.1371/journal.pone.0330910

**Published:** 2025-08-25

**Authors:** Sung-Min Son, Mo-Se Hwang, Yeon-Joo Nam, Kyu-Ho Jeong, June-Ho Seo, Tae Hui Kim

**Affiliations:** 1 Department of Psychiatry, Yonsei University Wonju Severance Christian Hospital, Wonju-si, Gangwon-do, Republic of Korea; 2 Department of Psychiatry, Yonsei University Wonju College of Medicine, Wonju-si, Gangwon-do, Republic of Korea; Taipei Veterans General Hospital, TAIWAN

## Abstract

**Backgrounds:**

Behavioral activation (BA) is recognized as an evidence-based therapy for depression and other mental health conditions. Using meta-analysis and systematic literature review to analyze the research trends is helpful, but it has limitations in understanding the flow and development of BA research.

**Purpose:**

This study aims to analyze BA research trends using keyword network analysis.

**Methods:**

The study examined network characteristics, centrality, and cohesion by conducting Keyword network analysis (KNA) on BA -related studies from 2003 to 2023. The research was divided into two periods: 2003–2012 and 2013–2023.

**Results:**

The analysis revealed an increase in the number of keywords from 26 in the first period to in the second, along with a rise in average degree and keyword links. Notably, cancer research surged during the second period. In terms of centrality, the maximum value rose in the second period, with “Depression” maintaining the highest centrality in both periods. For eigenvector centrality, “Older adult” ranked highest in the first period, while “Psycho-oncology” took the lead in the second. Cohesion analysis showed that in the first period, “Depression” was linked to older adult inpatient care, dealing with issues like “Complicated bereavement,” “Posttraumatic stress disorder,” and “Cancer,” as well as serving as a bridge for depression and smoking cessation. In the second period, the scope of “Depression” expanded to include older adult, university students, and children, with a focus on emotional variables, social support, and non-face-to-face interventions.

**Conclusions:**

This study sheds light on the evolving landscape of research on BA therapy, highlighting its continuous expansion in addressing depression across diverse populations and settings. Our study is the first to provide a comprehensive overview of the research keywords over the past two decades, offering a knowledge map and flow. This contributes to facilitating practical application in clinical settings and understanding research trends related to BA therapy.

## Introduction

Behavioral activation (BA) has a history dating back to the 1970s as a therapeutic approach aimed at effectively addressing activity limitations caused by cognitive impairments, physical dysfunction, behavioral constraints, and depression [1,2]. The primary goals of BA are to increase engagement in positively rewarding activities while reducing patterns of avoidance behaviors, ultimately aiming to alleviate mental health conditions and enhance overall functioning and quality of life [3–5]. BA reflects a trans-diagnostic approach, understanding the various symptoms and mechanisms present across different disorders [6]. Therefore, it has been applied to various age groups, ranging from children to adolescents, adults, and the elderly, dealing with a diverse range of conditions and symptoms [2,3,5,7–9]. As the COVID-19 pandemic has progressed, BA has begun to shift to digital approaches, and their utility has expanded further [10].

Studies on research trends are essential to effectively grasp the scope and content of research in the BA therapeutic approach field. Jacobson et al. [11] validated the fundamental concepts and effectiveness of the BA, advocating for its expansion. Such studies aid in understanding key trends and topics in the field, providing insights into emerging themes and future directions. Analyzing research trends is considered a key process for Evidence-Based Practice (EBP). Analyzing research trends provides evidence and value for optimal decision-making and serves as important foundational data to enhance the effectiveness of treatment approaches [3]. Therefore, research trend analysis helps to identify changes and trends in research topics within specific fields over time and reveals the evolving interests of researchers in the field. This contributes to providing academic value and significance within the field and holds practical applicability in real-world settings [12]. Particularly, various fields such as healthcare, rehabilitation, and psychology continuously report research analyzing trends related to specific topics, which are recognized as essential to EBP [3,13]. Furthermore, research trend analysis is also considered a crucial element of EBP in clinical settings. Clinical physicians and therapists constantly monitor research trends to incorporate the latest research findings and trends into patient care, enabling evidence-based decision-making in clinical practice [14].

In the analysis of research trends related to BA, literature review methods such as Meta-analysis and Systematic review were predominantly utilized [15]. Sturmey [16] conducted a Meta-analysis to investigate the clinical study status and effectiveness of BA. Kanter et al. [2] presented the mechanisms, processes, and components of BA through an empirical literature review. Dimidjian et al. [3], Dimaggio & Shahar [7], and Forbes [8] analyzed theoretical, experiential, and clinical components of BA by examining trends in methods and target populations. However, these studies often limited their scope to presenting the content of literature, making it challenging to understand the relationships and structures among the literature. Huguet et al. [17,18] utilized Systematic review to investigate trends in clinical studies, evaluate the quality of literature and evidence, and summarize relevant evidence. They analyzed the occurrence years and research methods of relevant literature and presented the characteristics and content of the research using indicators such as frequency and percentage. However, these studies have limitations in understanding the flow and changes in BA application due to their focus on characteristics reported over specific periods and their independent analysis of variables.

Existing review literature has often failed to consider various aspects of BA by focusing on specific topics. For example, studies by Ekers et al. [19] and Richards et al. [20] primarily emphasized the analysis of the effectiveness of BA and the impact on subgroups. Moreover, these studies mainly investigated the effectiveness and changes in depression treatment approaches, without including analysis of variables related to other psychological symptoms or functional outcomes. Consequently, there were limitations in understanding patient characteristics and the potential for generalization to various mental health issues or population groups. Moreover, various review literature has limitations in providing comprehensive analysis regarding the temporal constraints and trends. The study by Lejuez et al. [21] focused on the initial experimental research of BA but had limitations in terms of the research period. They did not provide information on how BA changes over time. Additionally, studies such as Cuijpers et al. [22] synthesized and analyzed the current research outcomes of BA but did not address temporal trends and changes. As a result, there were limitations in understanding the flow and development of BA research.

Keyword network analysis (KNA) is a methodology used to identify and understand topics or concepts by utilizing keywords specified by authors in research papers, and to grasp the relationships between these topics. It serves as an essential tool for historically documenting the development and changing research trends in a particular field. KNA is valuable for identifying key topics and visualizing interactions between them [23]. Additionally, it enables the quantification of relationships between reported keywords, facilitating a quantitative analysis of their relationships [24]. By employing a wide range of keywords, KNA allows for the identification of structural relationships between various concepts and visually represents these relationships to facilitate intuitive understanding of the relationships between keywords, with core keywords at the center [25]. Particularly, centrality analysis of keywords helps identify core keywords and understand their roles and influence [26], while cohesion structure analysis enables a structured understanding of relationships between keywords [24]. Such analyses are useful for understanding core topics, relationships between topics, and research trends in a field, as well as for suggesting new research directions and determining effective research methods [27].

KNA related to BA research will be instrumental in providing guidelines and directions for utilization in clinical settings and subsequent research. By identifying application items of BA therapeutic approach centered around core keywords, practical guidelines that can be implemented in actual treatment settings can be provided. Moreover, the results obtained through KNA can serve as the basis for recommendations for future research and can be utilized to explore new ideas. Nevertheless, there is currently no existing research trend analysis utilizing KNA to understand the progression of BA research overtime and derive practical guidance. Therefore, the purpose of this study is to analyze the trends in BA research over 20 years by utilizing KNA to comprehend the flows and influence of BA.

## Materials and methods

### Study subjects

The subject of this study is the keywords extracted from a total of 117 studies related to behavioral activation (including 8 literatures from Korea). To minimize bias in the process of keyword collection, a wide range of studies were searched using 2 Korean and 3 international databases. For the collection of keywords, searches for foreign literature utilized PubMed, PsycINFO, and MEDLINE, which are most used in behavioral activation-related review literature. For Korean literature, Research Information Sharing Service (RISS) and Korea Citation Index (KCI) were employed. The search keywords were set as “BA” or “Behavioral activation,” and after reviewing the titles and abstracts of relevant literature, the final literature was selected, and keywords contained in the literature were extracted.

### Inclusion and exclusion criteria

The criteria for selecting literature for this study are as follows: 1) Literature that includes the search terms “BA” or “Behavioral activation” in the title and keywords, and 2) Experimental research literature targeting humans. Exclusion criteria include: 1) Review literature, 2) Literature where BA therapeutic approach is included as a subcategory in treatment approaches such as CBT, ACT, 3) Grey literature not published in academic journals, 4) Theses, reports, books, and conference presentations, 5) Literature not in English or Korean, and 6) Literature outside the analysis period of this study. The search period was divided into two periods based on the reporting of BA therapeutic approach-related clinical studies starting from 2003, with each period spanning 10 years: Period 1 (2003–2012) and Period 2 (2013–2023) and analyzed accordingly.

### Data extraction and quality assessment

**Fig 1:**
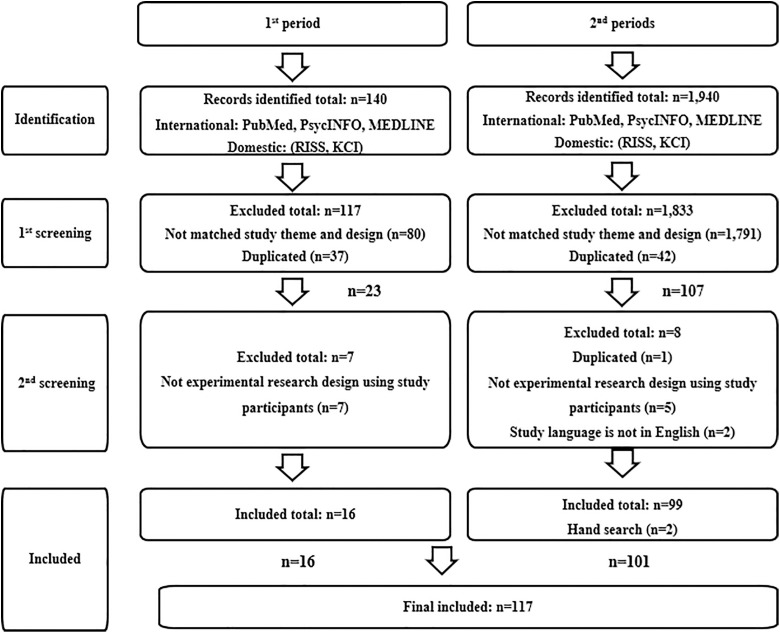
Flow chart of research inclusion.

The results of literature collection for this study are presented in (Fig 1). During Period 1, a total of 140 studies were retrieved, of which 80 were excluded due to mismatched research topics and designs after the first screening, along with 37 duplicate studies, resulting in the exclusion of 117 studies. Consequently, 23 studies were collected. Upon secondary review, 7 studies that were not experimental studies involving participants were excluded, leaving a total of 16 studies collected for Period 1 of this study. In Period 2, a total of 1,940 domestic and international studies were identified. Following the first screening, 1,791 items were excluded due to mismatched research topics and designs, along with 42 duplicate items, resulting in the exclusion of 1,833 studies. Subsequently, 107 studies were collected. Upon secondary review, 5 studies that were not experimental studies involving participants, 1 duplicate item, and 2 items not in English were excluded. Additionally, 2 studies were added through hand search, resulting in a final collection of 101 studies for Period 2 of this study.

### Study procedures

**Fig 2:**
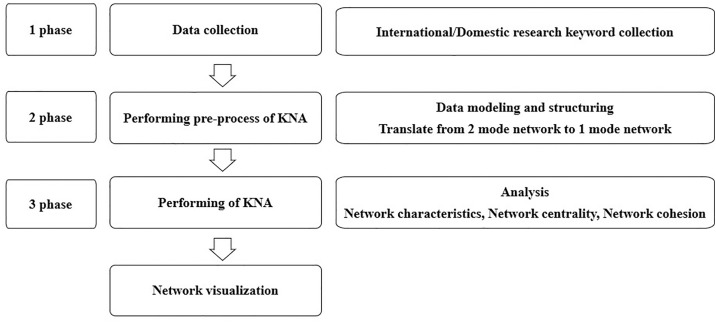
Keyword network analysis process.

To analyze trends in research related to behavioral activation, this study was conducted in three stages, as depicted in (Fig 2). The first stage involved data collection, during which keywords were gathered from the literature. Subsequently, in the second stage, preprocessing was conducted using the collected keywords to perform network modeling. This process involved structuring the relationships between keywords into a consistent format, transforming the 2 mode network of papers and keywords into a 1-mode network of independent individual keywords to enable separate analysis. The 1 mode network artificially establishes relationships between keywords, even when there is no direct relationship between them, to represent the relationships between keywords. In this process, the degree of connectivity between keywords was analyzed, and the frequency of appearance of each keyword in papers was calculated. Keywords with duplicate appearances were then removed based on their frequency, resulting in the conversion of the network into a matrix format. Finally, in the last stage, keyword network analysis was performed. Data collection and the KNA were conducted by three researchers involved in this study.

### Keyword network analysis

Keyword network analysis (KNA) was conducted using NetMiner 4.0 (Cyram, Co., Korea), which allowed for the analysis of network distribution and centrality metrics. Network distribution metrics describe the characteristics of the network structure, including the overall number of connections between keywords and the number of connections associated with each keyword. Specifically, the metrics analyzed included the number of keywords, the number of links, average degree, density, mean distance, diameter, inclusiveness, and the number of isolated keywords. Network centrality metrics express the degree to which a keyword is positioned at the center of the entire keyword network, indicating its importance and influence within the network, as well as its associations with other keywords [25]. Thus, centrality metrics, including in-degree/out-degree centrality, keyword betweenness centrality, and eigenvector centrality, were analyzed to identify keywords with high occurrence frequencies that play central roles in behavioral activation-related research, ranked according to their significance.

Centrality metrics are crucial indicators explaining the significant position of a node within an entire network, indicating outstanding leadership, popularity, or excellent reputation [28]. Social actors with higher centrality are positioned at the core of the network, acquiring greater power, influence, and convenience through the network [29]. Degree centrality describes the structural position within a network based on the number of connections, where individuals with the most connections occupy the network’s center [30]. In a keyword network, degree centrality represents the most connected keywords, indicating the most frequently mentioned keywords [26]. Betweenness centrality measures how well a node connects different individuals, indicating the extent to which it acts as a mediator or intermediary. Individuals performing the most intermediary roles are positioned at the network’s center [28]. In a keyword network, betweenness centrality identifies keywords that mediate relationships between various other keywords [26]. Eigenvector centrality considers the centrality of connected individuals, where nodes connected to influential individuals are positioned at the center of the network [30]. In a keyword network, eigenvector centrality reflects how frequently a keyword is mentioned alongside influential keywords [26].

Coherence analysis examines the strength and close relationships among individual keywords within the network, revealing the structure of relationships among various keywords in the network. This analysis helps understand how keyword groups are formed and the relationships between them [22–25].

## Results

### Results of network characteristics

The results of the network characteristics are presented in Table 1. In the first period, the total number of keywords, excluding duplicates, was 26, with a total of 38 connections between keywords. The average degree of individual keywords was 3.040, and the density was .127, indicating a connectivity degree of 12.7% relative to the total connections. The average distance between keywords was 2.243, and the diameter of the entire network was 4. The inclusiveness was 1 (100%), with no isolated keywords observed, indicating that all keywords were interconnected.

**Table 1:** Results of network characteristics.

Items	Period 1 (03-12)	Period 2 (13-23)	Difference (Period 2−1)
Number of keywords (n)	26	151	+125
Number of link (n)	38	702	+664
Average degree (n)	3.040	9.298	+6.258
Density (%)	.127 (12.7)	.062 (6.2)	−.065
Mean distance (values)	2.243	2.159	−.084
Diameter (values)	4	4	0
Inclusiveness (%)	1 (100)	1 (100)	0
Isolated keywords (n)	0	0	0

In the second period, the total number of keywords, excluding duplicates, increased to 151, with a total of 702 connections between keywords. The average degree of individual keywords was 9.298, and the density was .062, indicating a connectivity degree of 6.2% relative to the total connections. The average distance between keywords was 2.159, and the diameter of the entire network remained 4. The inclusiveness was 1 (100%), with no isolated keywords observed, indicating that all keywords were interconnected.

Compared to the first period, the second period saw an increase in both the total number of keywords by 125 and the total number of connections by 664. The average degree of connectivity also increased by 6.258. However, the density decreased to .065, representing a 6.5% decrease, and the average connection distance decreased by .084. This was a result of the increase in the number of keywords and connections. The diameter of the entire network, inclusiveness, and the number of isolated keywords remained the same.

### Results of network centrality

**Fig 3:**
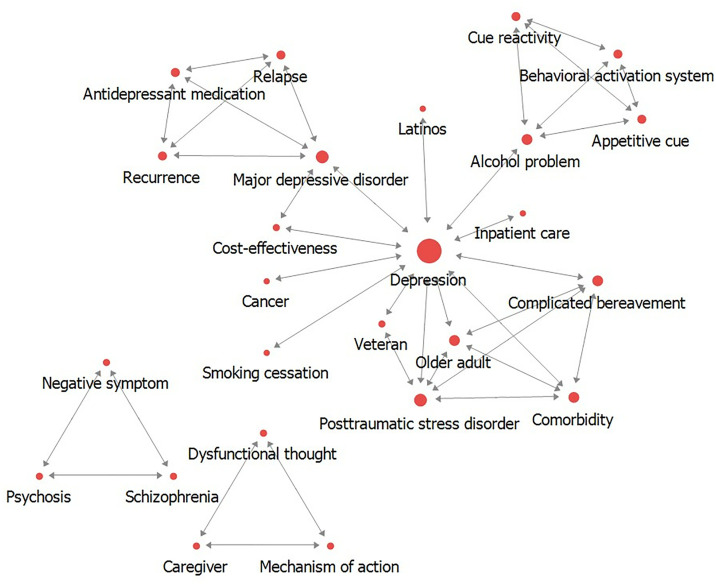
Results of network centrality for period 1.

The results of centrality analysis are presented in Table 2. In terms of degree centrality, both inward and outward degree centrality values were found to be identical. In the first period, the degree centrality exhibited an average of .127, with a maximum of .500 and a minimum of 0.042. The keyword betweenness centrality had an average of .032, with a maximum of .466 and a minimum of 0. Eigenvector centrality showed an average of .106, with a maximum of .464 and a minimum of 0 (Fig 3).

**Table 2:** Results of descriptive analysis of network centrality.

Items	Period 1 (03–12 yrs)		Period 2 (13–23 yrs)	
	Mean ± SD	Max/ Min	Mean ± SD	Max/ Min
In/Out degree centrality (values)	.127 ± .090	.500/ 0.042	.062 ± .078	.747/.007
Keyword betweenness centrality (values)	.032 ± .099	.466/ 0	.006 ± .046	.559/ 0
Eigenvector centrality (values)	.106 ± .169	.464/ 0	.035 ± .073	.243/ 0

**Fig 4:**
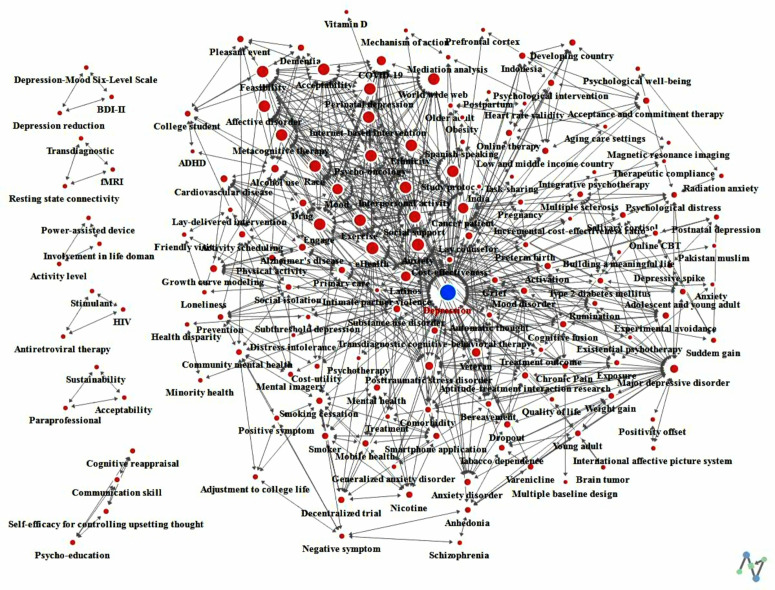
Results of network centrality for period 2.

In the second period, the degree centrality had an average of .062, with a maximum of .747 and a minimum of 0. Keyword betweenness centrality showed an average of .006, with a maximum of .559 and a minimum of 0. Eigenvector centrality exhibited an average of .035, with a maximum of .243 and a minimum of 0 (Fig 4).

Comparing the first and second periods, the average values of degree centrality and keyword betweenness centrality decreased in the second period, while the maximum values increased. Eigenvector centrality showed a decrease in both average and maximum values.

#### Results of in/out degree centrality by period.

**Fig 5:**
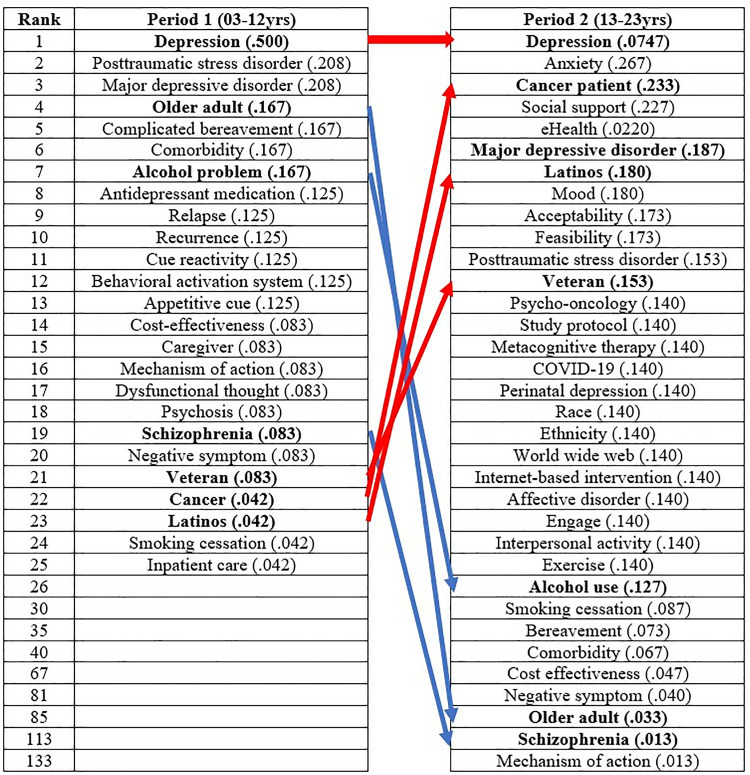
Results of in/out degree centrality by period.

The results of degree centrality are presented in Fig 5. Both inward and outward degree centrality revealed that the same keywords had high centrality. In the first period, the top 25 keywords with the highest degree centrality were identified. Among them, “Depression” (.500) exhibited the highest centrality, followed by “Posttraumatic stress disorder,” “Major depressive disorder,” “Older adult,” and “Complicated bereavement.” In the second period, to demonstrate the change in the number of keywords from the first period, the same keywords as the first period were included. The keyword with the highest degree centrality in the second period was “Depression” (.747), followed by “Anxiety,” “Cancer patient,” “Social support,” and “eHealth.” Keywords that increased in rank from the first to the second period included “Cancer patient” (22nd to 3rd), “Latinos” (22nd to 7th), and “Veteran” (21st to 12th). Keywords that experienced a significant decrease in rank included “Older adult” (4th to 85th), “Alcohol problem” (7th to 26th), and “Schizophrenia” (19th to 113th).

#### Results of keyword betweenness centrality by period.

**Fig 6:**
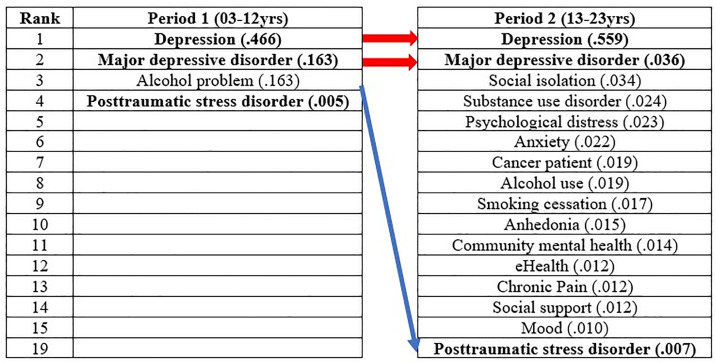
Results of node betweenness centrality by period.

The results of betweenness centrality are presented in Fig 6. In the first period, a total of four keywords exhibited high betweenness centrality. Among them, “Depression” (.466) showed the highest centrality, followed by “Major depressive disorder,” “Alcohol problem,” and “Posttraumatic stress disorder.” Considering the keyword weight compared to the first period, a total of 15 keywords were listed in the second period, including the same keywords as the first period. Among them, “Depression” (.559) had the highest betweenness centrality, followed by “Major depressive disorder,” “Social isolation,” and “Substance use disorder.” A keyword that experienced a significant decrease in rank from the first to the second period was “Posttraumatic stress disorder” (4th to 19th).

#### Results of eigenvector centrality by period.

**Fig 7:**
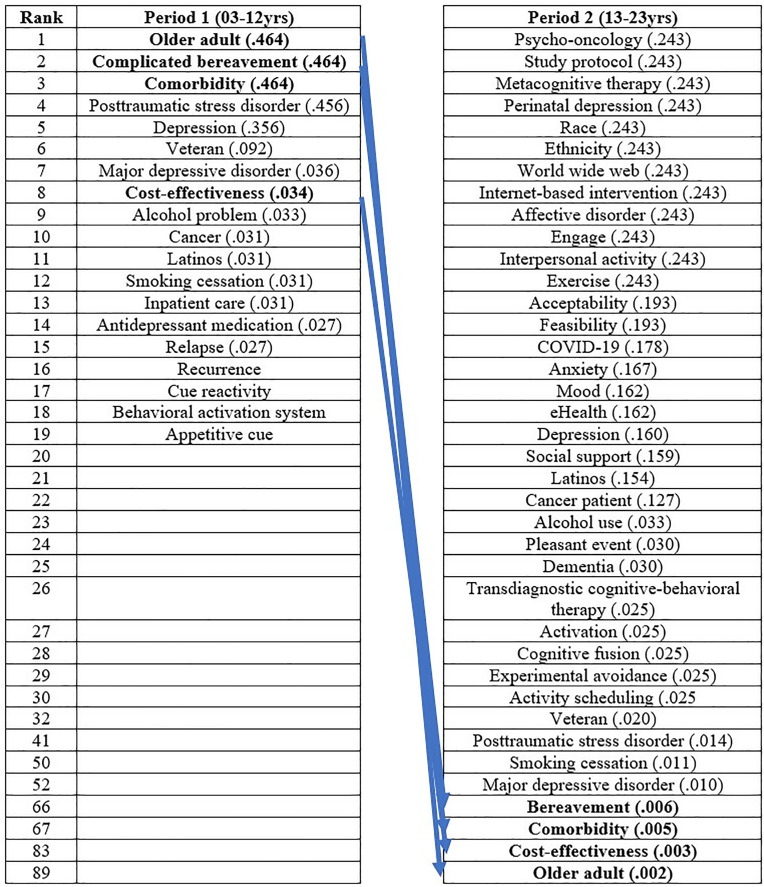
Results of eigenvector centrality by period.

The results of eigenvector centrality are presented in Fig 7. In the first period, the keywords with the highest eigenvector centrality were listed up to the 19th rank. The keyword with the highest eigenvector centrality in the first period was “Older adult” (.462), followed by “Complicated bereavement,” “Comorbidity,” “Posttraumatic stress disorder,” and “Depression.” In the second period, the keyword with the highest eigenvector centrality was “Psycho-oncology” (.243), followed by “Study protocol,” “Metacognitive therapy,” “Perinatal depression,” and “Race.” Keywords that experienced a significant decrease in rank from the first to the second period were “Older adult” (1st to 89th), “Complicated bereavement” (2nd to 66th), “Comorbidity” (3rd to 67th), and “Cost-effectiveness” (8th to 83rd).

### Results of network cohesion

**Fig 8:**
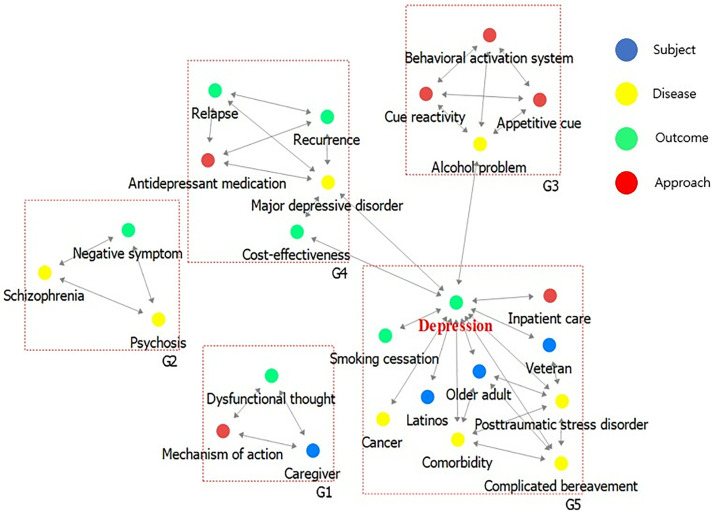
Results of network cohesion analysis for period 1.

**Fig 9:**
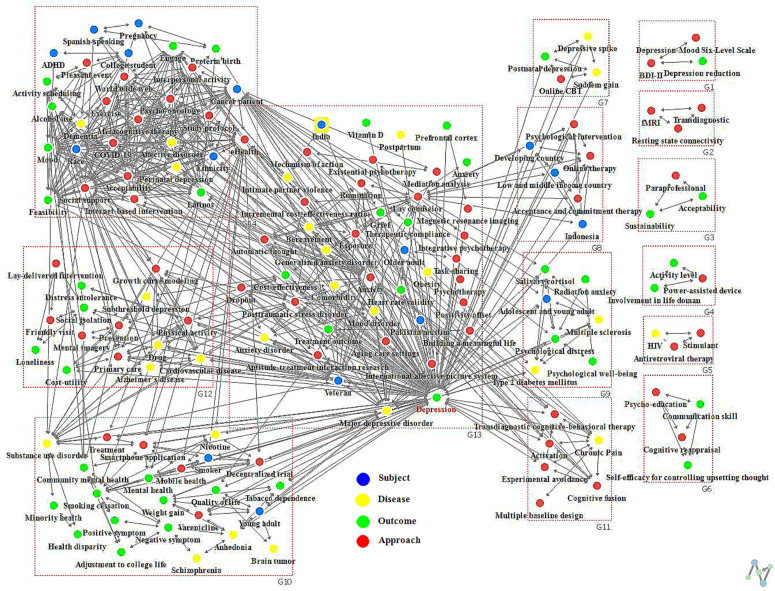
Results of network cohesion analysis for period 2.

The results of cohesion structure are presented in Table 3 and (Fig 8) for the first period, and Table 4 and (Fig 9) for the second period. In the first period, a total of 5 communities were identified with a Modularity value of .623 (62.3%). Groups 1 and 2 contained 3 keywords each, while Group 3 included 4 keywords. Group 4 comprised 5 keywords, and Group 5 had 10 keywords. In the second period, a total of 14 communities were identified with a Modularity value of .465 (46.5%). Groups 1–7 contained 3 keywords each, Groups 8 and 11 contained 6 keywords each, Group 9 contained 7 keywords, and Groups contained 23, 15, 42, and 29 keywords, respectively.

**Table 3:** Results of network cohesion analysis for period 1.

**Items**	Period 1 (03–12 yrs)
**Number of** **communities (n)**	5
**Modularity (value, %)**	.623 (62.3)
**Group 1 (n = 3)**	Subject: Caregiver, Outcome: Dysfunctional thought Approach: Mechanism of action
**Group 2 (n = 3)**	Disease: Schizophrenia, Psychosis Outcome: Negative symptom
**Group 3 (n = 4)**	Disease: Alcohol problem, Approach: Cue reactivity, Behavioral activation system, Appetitive cue
**Group 4 (n = 5)**	Disease: Major depressive disorder Outcome: Cost-effectiveness, Relapse, Recurrence Approach: Antidepressant medication
**Group 5 (n = 10)**	Subject: Older adult, Latinos, Veteran Disease: Complicated bereavement, Posttraumatic stress disorder, Comorbidity, Cancer Outcome: Depression, Smoking cessation Approach: Inpatient care

**Table 4:** Results of network cohesion analysis for period 2.

**Items**	Period 2 (13–23 yrs)
**Number of communities (n)**	14
**Modularity (value, %)**	.465 (46.5)
**Group 1 (n = 3)**	Outcome: Depression reduction Approach: BDI-Ⅱ, Depression-Mood Six-Level Scale
**Group 2 (n = 3)**	Approach: fMRI, Transdiagnostic, Resting state connectivity
**Group 3 (n = 3)**	Outcome: Acceptability, Sustainability Approach: Paraprofessional
**Group 4 (n = 3)**	Outcome: Activity level, Involvement in life domain Approach: Power-assisted device
**Group 5 (n = 3)**	Disease: HIV Approach: Antiretroviral therapy, Stimulant
**Group 6 (n = 4)**	Outcome: Self-efficacy for controlling upsetting thought, Communication skill Approach: Psycho-education, Cognitive reappraisal
**Group 7 (n = 4)**	Disease: Depressive spike, Sudden gain Outcome: Postnatal depression Approach: Online CBT
**Group 8 (n = 6)**	Subjects: Low- and middle-income country, Indonesia, Developing country Approach: Psychological intervention, Online therapy, Acceptance and commitment therapy
**Group 9 (n = 7)**	Subject: Adolescent and young adult Disease: Multiple sclerosis, Type 2 diabetes mellitus Outcome: Psychological distress, Salivary cortisol, Radiation anxiety, Psychological well-being
**Group 10 (n = 23)**	Subject: Smoker, Young adult Disease: Nicotine, Brain tumor, Schizophrenia, Substance use disorder, Anhedonia Outcome: Weight gain, Smoking cessation, Mental health, Quality of life, Tobacco dependence, Negative symptom, Health disparity, Minority health, Adjustment to college life, Positive symptom Approach: Mobile health, Smartphone application, Decentralized trial, Varenicline, Treatment, Community mental health
**Group 11 (n = 6)**	Disease: Chronic Pain Approach: Transdiagnostic cognitive-behavioral therapy, Activation, Cognitive fusion, Experimental avoidance, Multiple baseline design
**Group 12 (n = 15)**	Disease: Drug, Subthreshold depression, Cardiovascular disease, Alzheimer’s disease Outcome: Social isolation, Loneliness, Distress intolerance, Cost-utility Approach: Prevention, Mental imagery, Physical activity, Friendly visit, Lay-delivered intervention, Primary care, Growth curve modeling
**Group 13 (n=42)**	Subject: Pakistan Muslim, Older adult, Veteran, IndiaDisease: Major depressive disorder, Generalized anxiety disorder,Bereavement, Intimate partner violence, Postpartum, Obesity,Posttraumatic stress disorder, Comorbidity, Mood disorder,Anxiety disorderOutcome: Depression, Anxiety, Heart rate validity, Treatmentoutcome, Cost-effectiveness, Grief, Vitamin D, Prefrontal cortex,Therapeutic complianceApproach: Psychotherapy, Exposure, Incremental cost-effectivenessratio, Lay counselor, Aptitude-treatment interactionresearch, Dropout, Building a meaningful life, Task-sharing,Rumination, Automatic thought, Mediation analysis, Mechanismof action, Existential psychotherapy, Integrative psychotherapy,Positivity offset, International affective picture system, Magnetic resonance imaging
**Group 14 (n=29)**	Subject: Pregnancy, Cancer patient, College student, ADHD,Latinos, Spanish-speaking, Race, EthnicityDisease: Perinatal depression, Dementia, Affective disorderOutcome: Preterm birth, Alcohol use, Acceptability, Feasibility,Mood, EngageApproach: Social support, eHealth, Psycho-oncology, Studyprotocol, Metacognitive therapy, Activity scheduling, Pleasantevent, World wide web, Internet-based intervention, Interpersonal activity, Exercise, COVID-19

## Discussion

### Strength of this study

This study analyzed trends in research related to BA using KNA, thereby presenting a new perspective and approach. The strengths of this study are as follows.

Firstly, this study conducted research by considering various aspects and their relationships. To achieve this, it utilized KNA to analyze the structure of keywords and provide a related knowledge map based on the relationships formed by these keywords. This allowed for a clearer understanding of the relationships between various topics and concepts, overcoming the limitations of previous studies that focused solely on the effectiveness of therapeutic approaches for specific topics or targets. Moreover, it presented a more comprehensive perspective by including analysis of variables related to various psychological symptoms or functional outcomes. Such analysis plays a crucial role in ensuring the comprehensiveness of the research and holds significant importance in understanding the mutual characteristics of relevant topics and concepts. Borgatti et al. [31] mentioned that applying KNA in trend analysis within research helps in understanding the connectivity of keywords and the relevance of significant topics and concepts within a specific field, thus aiding in comprehending changes and expansions in the research field as a whole. This observation underscores the contribution of this study to understanding the changes and advancements in the relevant field.

Secondly, this study comprehensively analyzed previous research over the past two decades based on the historical trajectory of BA therapeutic approach, enabling a comprehensive understanding of the development and trends of BA therapeutic approach [21]. Through this, the study adopted a more structured and systematic approach by utilizing KNA to understand the development and changes of BA therapy approach. Furthermore, it contributed to a better understanding of the research trends regarding BA therapeutic approach compared to previous studies. Kim & Hastak [32] also reported that the application of KNA analysis provides important insights into understanding the structural development and changes of BA therapeutic approach. These studies emphasize the contextual roots of the concept and history of BA therapeutic approach, highlighting the importance of analyzing theoretical concepts. Therefore, the results of this study can be evaluated as valuable evidence for understanding the changes and development of BA therapeutic approach through the application of KNA, serving as important evidence for understanding the development and future directions of BA therapeutic approach.

### Discussion of study results

The purpose of this study is to analyze the research trends in BA utilizing KNA to understand its development and trajectory. Through this, the study aimed to evaluate the diversity and expansiveness of BA research. As a result, significant differences in research trends between period 1 (2003–2012) and period 2 (2013–2023) were identified, indicating an expansion in the scope and content of BA. While period 1 was predominantly centered around traditional depression treatment, period 2 showed an emergence of keywords related to social and cultural contexts, suggests an expansion of research to consider various psychological issues and symptoms.

BA is recognized as a primary therapeutic approach utilized to improve patients’ mental health, garnering attention as a third-wave cognitive-behavioral therapeutic approach that focuses on changes in context and experience [33]. BA therapeutic approach goes beyond the paradigms presented in the past within the mental health field, offering a new perspective on addressing patients’ symptoms by emphasizing individual relationships and contextual characteristics [34]. Given these premises, understanding the evolution of treatment methodologies since the 2000s, as proposed by BA, is crucial both academically and clinically. Based on these findings, this study provides several valuable insights below.

Firstly, it reveals a growing number of studies on the BA, indicating its versatility in addressing diverse population and symptoms. This suggests an expansion in the scope and methodologies of BA research. These findings indicate that the BA therapeutic approach is gradually evolving, and its scope is becoming more comprehensive, supported by various studies. Furthermore, the results of this study confirm the increasing recognition of the importance of the BA therapeutic approach. Specifically, it demonstrates the growing utilization of BA as a third-wave cognitive behavioral therapeutic approach, emphasizing the consideration of individual surroundings and characteristics to enhance therapeutic effects. Thus, this study illustrates the progressive trend in BA and offers the potential to provide patients with better treatment options. Verifying the basic concepts and effectiveness of BA is important for the expansion of BA [11]. Our study findings serve as empirical evidence supporting the assertion made by individual researchers that the scope and depth of BA application will continue to expand.

Secondly, through the analysis of KNA results, it was observed that the BA is expanding as a comprehensive approach to various symptoms. This indicates a departure from the traditional disease-centered approach, as therapeutic interventions are being applied not only to depression but also to various psychological issues and symptoms. Such an approach reflects a trans-diagnostic approach, understanding the various symptoms and mechanisms present across different disorders [6]. In the analysis results, it was noted that research focusing on key targets such as “Older adult,” “Alcohol problem,” and “Schizophrenia” is increasing, alongside a rise in studies related to previously less emphasized targets. Particularly noteworthy is the prominence of “Cancer patient” as an influential keyword among these targets, indicating an expansion of research scope to include diverse populations such as “Latinos” and “Veteran.” Moreover, there is a need to investigate variables related to psychological issues and symptoms and expand research on the therapeutic effects of BA based on these variables. Through this, it is anticipated that the BA therapeutic approach will further evolve as an effective treatment for various psychological difficulties.

Particularly, the prominence of keywords such as “Social support,” “Engage,” and “Interpersonal activity” in the analysis results indicates an increasing focus on exploring the applicability of the BA in various social and cultural contexts. This expansion is perceived as a result of adopting an approach that considers diverse psychological, social, and physiological factors, understanding the relationships between various symptoms and behaviors without being confined to depression or specific mental disorders. Such changes are deemed to provide insights into therapeutic approaches for addressing diverse conditions and symptoms. Therefore, the findings of this study are considered to contribute to improving the course of illness and treatment outcomes for patients in the mental health field. Furthermore, it is anticipated that approaches in these social and cultural contexts will play a vital role in developing and implementing tailored treatment methods for local communities. Subsequent research should delve deeper into the effectiveness of the BA therapeutic approach in social and cultural contexts and develop approaches suitable for various local communities. Such studies can serve as valuable references for the development of policies and programs aimed at promoting patients’ mental health.

Thirdly, the importance of digital approaches has been emphasized amidst the COVID-19 pandemic [10]. This is evident in the analysis results of this study, where keywords such as “eHealth,” “Worldwide web,” and “Internet-based intervention” were prominent. The prominence of keywords related to digital approaches underscores the significance of utilizing digital technologies in BA therapeutic approaches. This highlights the importance of employing digital technologies in BA therapeutic approach, including the development and utilization of remote therapy and counseling, as well as online platforms for intervention services. Such utilization is deemed crucial for applying BA therapeutic approach for mental health promotion among populations facing limitations in mobility and accessibility. Based on this, research related to BA therapeutic approach should focus on providing therapeutic services utilizing digital technologies and verifying their effectiveness. Exploratory studies investigating the applicability of digital approaches are also warranted. Through these efforts, the advancement of BA therapeutic approach through digital means is expected to serve as an important tool for promoting mental health at both individual and group levels.

Lastly, the analysis of cohesion structure and centrality results reveals a clear evolution of research related to the BA therapeutic approach between Period 1 and Period 2, indicating a convergent development. From Period 1 to Period 2, there was an increase in the diversity and comprehensiveness of research on the BA therapeutic approach, as evidenced by the formation of keyword relationship structures centered around traditional depression treatment and specific target applications in Period 1. However, in Period 2, social-cultural contextual keywords emerged, indicating the expansion of BA’s therapeutic approach to consider various psychological issues and symptoms. This trend was further confirmed by the increase in convergence and interaction among various keywords. Utilizing the results of this study, it is anticipated that BA research will involve convergent studies considering various domains and characteristics grouped into bundles of keywords. Furthermore, establishing an integrative approach in BA therapeutic approach-related research, focusing on the communities formed by keywords, can be pursued. Based on the studies by Kang et al. [12] and Ko [14], this study’s findings can be utilized in real clinical settings to enhance the comprehensive and effective utilization of the BA therapeutic approach.

As a result of the analysis, BA was found to be adaptable for diverse populations and a wide range of conditions, including depression, anxiety, and comorbid chronic illnesses. This adaptability stems from its structured yet flexible approach, which allows clinicians to tailor interventions to individual patient needs. From a methodological perspective, this study highlights the importance of systematically identifying key components of BA that can be customized based on factors such as cultural context, symptom severity, and patient preferences. For instance, integrating digital tools to deliver BA remotely can enhance accessibility for patients with mobility limitations or those living in underserved areas. From a clinical perspective, the findings provide actionable insights for designing patient-centered interventions, such as leveraging activity monitoring to establish baseline behaviors and setting achievable activation goals to sustain engagement. The study also underscores the value of incorporating psychoeducation and collaborative goal-setting as foundational elements in BA delivery. These findings contribute to evidence-based practice by serving as a guideline for clinicians to not only expand the scope of BA to diverse populations but also refine its content to ensure measurable and sustainable outcomes. By recognizing BA’s flexibility and its application across various clinical scenarios, practitioners can enhance its effectiveness in real-world settings.

### Study limitations

This study has various limitations and constraints. Firstly, the data used in this study were collected from a specific academic database, which may result in the limited scope of the collected data. Additionally, only literature published in English and Korean was included, potentially excluding relevant literature published in other languages. Furthermore, unpublished literature, theses, reports, and academic conference presentations were not considered in the analysis. Therefore, caution is needed when interpreting the results of this study. Secondly, this study conducted a comprehensive analysis of the trends in BA therapeutic approach over a period of 10 years. However, this division may have limitations in capturing the detailed status and changes of BA therapeutic approach based on policy applications, social changes, specific situations, etc. Therefore, subsequent studies should consider conducting time-series analyses and analyses based on specific time points. Thirdly, while this study conducted a comprehensive analysis related to BA therapeutic approach, it has limitations in capturing the trends of specific variables and scales used. Thus, subsequent studies need to consider research that addresses these aspects.

## Conclusions

This study investigated recent trends and development in BA research using KNA, departing form conventional methods such as meta-analysis or systemic reviews. The analysis of research trends in BA, divided into 10-year time periods and examined for network characteristics and centrality, revealed that the BA is gradually evolving towards considering various domains and symptoms. Particularly, approaches that consider social and cultural contexts indicate the potential for more comprehensive and effective treatment methods. These research findings validate the diversity and expansiveness of BA therapeutic approach, laying the groundwork for more effective utilization in clinical settings. Furthermore, the utilization of KNA and analysis of keywords data structures have played a crucial role in structurally understanding the development and changes in BA therapeutic approach. It is expected that this will be recognized as important evidence for providing new knowledge in the field of mental health and suggesting future research directions. This study contributes to a clearer understanding of the development and changes in BA therapeutic approach, providing new insights into the field of mental health and offering potential for improving treatment outcomes.
